# Raman Spectroscopy and Related Techniques in Biomedicine

**DOI:** 10.3390/s100301871

**Published:** 2010-03-09

**Authors:** Andrew Downes, Alistair Elfick

**Affiliations:** School of Engineering, The University of Edinburgh, Edinburgh EH9 3JL, UK; E-Mail: alistair.elfick@ed.ac.uk

**Keywords:** Raman spectroscopy, CARS microscopy, Raman imaging

## Abstract

In this review we describe label-free optical spectroscopy techniques which are able to non-invasively measure the (bio)chemistry in biological systems. Raman spectroscopy uses visible or near-infrared light to measure a spectrum of vibrational bonds in seconds. Coherent anti-Stokes Raman (CARS) microscopy and stimulated Raman loss (SRL) microscopy are orders of magnitude more efficient than Raman spectroscopy, and are able to acquire high quality chemically-specific images in seconds. We discuss the benefits and limitations of all techniques, with particular emphasis on applications in biomedicine—both *in vivo* (using fiber endoscopes) and *in vitro* (in optical microscopes).

## 1. Introduction

Optical microscopy is highly valuable to biomedical research. White light microscopy reveals changes in refractive index within cells and tissue, but lacks any kind of biochemical contrast and is only appropriate for monolayers of cells or thin (<10 μm) sections of tissue as it does not have a 3D sectioning ability. Fluorescence imaging is the current major technique of interest. This relies on the use of fluorescent dyes which are either expressed by genes which have been modified, or are tagged on to molecules of interest. The drawbacks of fluorescence imaging are numerous: firstly, sample modification is required by some kind of labeling; secondly, this label of size 1–5 nm can perturb the behaviour of the tagged molecule of interest; thirdly, photobleaching limits the observation time and prevents long term studies.

Raman spectroscopy is a totally non-invasive, label-free technique which excites vibrations of molecular bonds. Although infrared spectroscopy can excite these vibrational bonds, it relies on low power sources and noisy detectors. Moreover, the wavelengths employed are in the infrared range (3–15 μm) so cannot produce high resolution images of cells which are of the order of 10 μm in diameter. Infrared spectroscopy also suffers from an extremely low penetration depth in aqueous solution, so is not applicable in live cells unless attenuated total reflection is used to probe a shallow depth above the substrate [1]. Synchrotron sources offer vastly improved spectral acquisition times—1,000 times faster [2] than benchtop FTIR—but it is not clear whether this is applicable to live cells, as heating by absorption may prevent any increase in acquisition speed.

**Figure 1.**
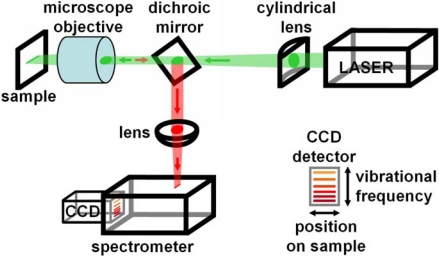
Schematic of Raman spectrometer. The displayed set-up focuses the illuminating laser (coloured green) down to a line on the sample (slit scanning mode), which can be replaced by a spot by removing the cylindrical lens. When a spot is illuminated at the sample, the Raman-shifted light (colored red) is filtered out from the laser light by a dichroic mirror, and dispersed along a vertical line on the two dimensional CCD detector. In slit-scanning mode, many spectra are acquired simultaneously: each position along the line on the sample produces a spectrum along the CCD detector.

Raman spectroscopy typically uses a laser wavelength of 532 nm (diode-pumped frequency doubled Nd:V) or 785 nm (AlGaAs diode), so can achieve a lateral resolution of better than half the wavelength (250–350 nm). This sub-cellular resolution is similar to that achieved in fluorescence imaging, and is far superior to the minimum resolution (∼0.1–10 mm) achievable with medical diagnostic techniques such as ultrasound, Magnetic Resonance Imaging, Positron Emission Tomography, or x-ray imaging. Some laser light which has lost energy after exciting one of the molecular vibrations is then red-shifted to a lower energy. This light is passed through a spectrometer, which disperses the light into a spectrum which is recorded with a cooled CCD camera. A schematic of the apparatus is shown in Figure 1. Live cells can easily be studied in cell culture media, on a heated stage on an inverted microscope linked to a Raman spectrometer. Such a system requires both a high collection efficiency and a high resolution spectrometer/CCD combination. It is highly beneficial to acquire a full high resolution Raman spectrum in one ‘shot’, so a CCD camera with a large lumber of pixels (>1,000) in the spectral axis is preferred.

The difference between the frequency of the incident laser light and that of the red-shifted light, is equal to the frequency of the vibrational bond which has been excited. Each molecule has a unique ‘fingerprint’ of Raman peaks at well defined frequencies [3] as biomolecules contain a variety of molecular bonds (e.g., C-H, C=C, O-H, aromatic ring) which are all excited. The spectrum senses the chemistry of the molecule(s) of interest, rather than performing elemental analysis—given that the information arises from the chemical bonds rather than from the nucleus. Frequency shifts are recorded in wavenumbers (cm^−1^), and biological molecules have vibrations in the range ∼600–3,000 cm^−1^.

**Figure 2.**
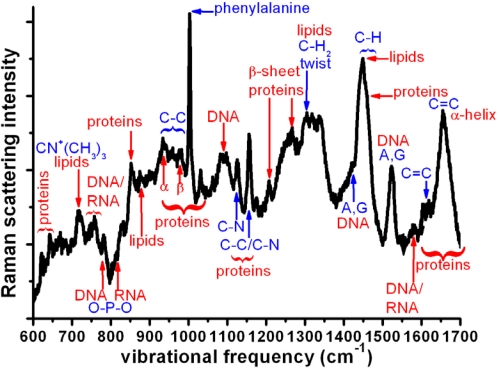
Unprocessed Raman spectrum of live MCF-7 breast cancer cells. 300 seconds acquisition time, 785 nm illumination, approximately 100 mW illumination power.

An example Raman spectrum from a single live cell is displayed in Figure 2. The cell—hence the spectrum—contains a very large number of different molecules, which fall into the four major groups of nucleic acids, proteins, lipids and carbohydrates. The complex chemistry of the cell is projected into the Raman spectrum, and subtle changes to their function (hence the biochemistry) results in changes to this spectrum. Hence, Raman spectroscopy is a sensitive measure of biochemistry within live cells, tissue and bone.

A single spectrum can be acquired from a small sub-cellular volume (0.01–0.04 μm^3^, depending on laser wavelength) or averaged over a larger area. A single spectrum is typically acquired in the range 1–10 s. A shorter wavelength gives faster acquisition, as the scattering co-efficient depends inversely on the fourth power of the laser wavelength. But shorter wavelengths are absorbed more strongly than longer wavelengths—heating of <1 °C has been measured directly in cells using 100 mW of 1,064 nm radiation in an optical trap [4]. The integrity of cells was confirmed for more than an hour’s exposure to 115 mW laser power at 785 nm [5], but morphological changes occurred in cells with 488 nm and 514.5 nm excitation at only 5 mW laser power [6]. The trade-off between signal strength (from the scattering co-efficient) and heating results in the balance for optimum signal lying in the laser excitation range 532–785 nm. In fact, lasers of sufficient power do not lie inbetween this range, so one or the other of these laser wavelengths is preferred.

**Figure 3.**
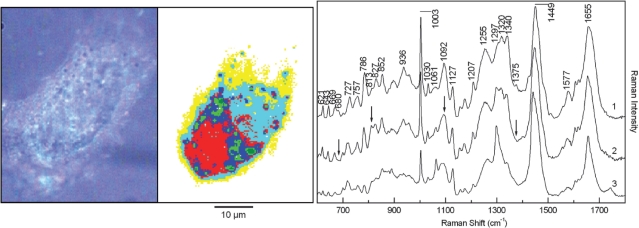
Photomicrograph of formalin-fixed lung fibroblast cell in buffer (left), Raman image after segmentation by cluster analysis (middle), Raman spectra (right) representing the nucleus (trace 1: red cluster), the cytoplasm (trace 2: cyan cluster) and lipid vesicles (trace 3: green cluster). © The Royal Society of Chemistry.

**Figure 4.**
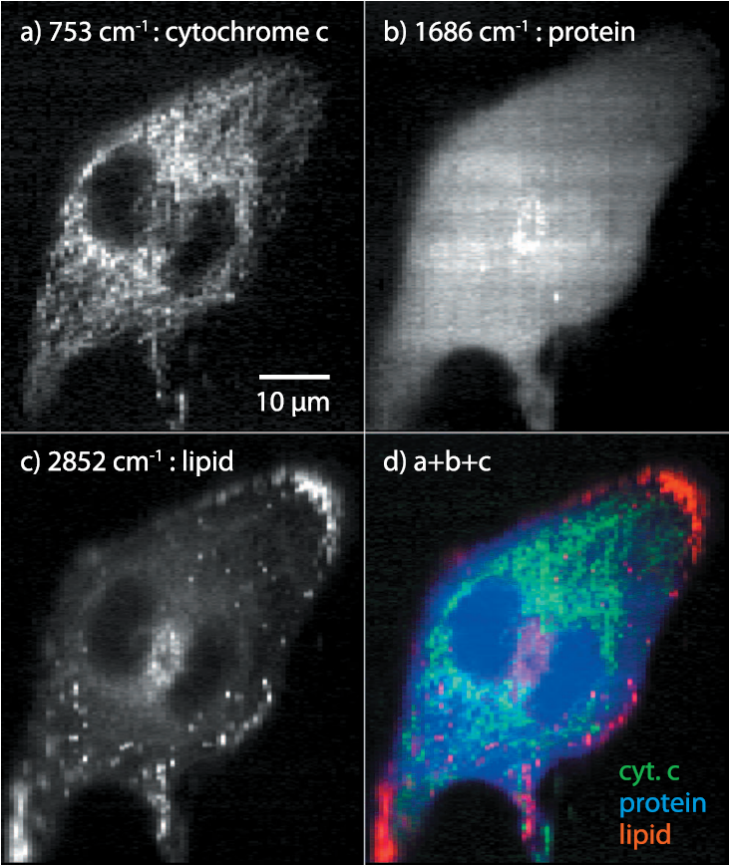
Raman scattering images of unstained, unlabelled living HeLa cells reconstructed using the distribution of Raman signals at (a) 753 cm^−1^, (b) 1,686 cm^−1^, and (c) 2,852 cm^−1^, showing the distribution of cytochrome c, protein beta sheet, and lipid molecules, respectively. Image (d) was constructed by merging images (a) through (c) with color channels. The sample was irradiated with a light intensity of 3.3 mW/μm^2^ at the focal plane in 78 lines of exposure. The exposure time of each line was 5 s, and the images consist of 78 × 281 pixels. © Society of Photo-Optical Instrumentation Engineers.

Although a few seconds would not be considered a long acquisition time for a single spectrum, it is sometimes necessary to average a large number of spectra acquired from an array of points over an area of cells or tissue. Equivalently, to build up an image by acquiring a spectrum at each scanning pixel would require many hours even for a basic 100 × 100 pixel image. This is too slow for live cell imaging—movement of the cell would blur out features, but fixed cells can easily be imaged like the one in Figure 3 [7], with laser powers which would cause damage to live cells (532 nm laser, 0.2 seconds per pixel, 120 × 150 pixels). This cannot greatly improved and is limited by the low Raman efficiency: only 1 in 10^8^–10^10^ incident photons is Raman-shifted. ‘Slit scanning’ Raman microscopy is able to reduce the time required for an image to a few minutes, by the parallel acquisition of spectra. In Figure 1, if a cylindrical lens is used, the laser focal spot on the sample is converted to a line. All points along the line relate to a specific column on the CCD chip; so a series of spectra corresponding to points along the line illuminated on the sample is recorded in a single CCD image. This process has very recently been used to acquire Raman images of live cells [8], and a slit-scanning image of live HeLa cervical cancer cells is shown in Figure 4 [9]: using 532 nm illumination, the image was acquired in 6.5 minutes. Cell cultures are typically grown on a plastic or glass substrate, but plastic is a dense source of C-C, C-H and other bonds present in cells, and glass gives rise to fluorescence which dominates over the Raman signal. So a crystalline substrate such as quartz, MgF, or CaF_2_ is essential ‘*in vitro*’.

**Figure 5.**
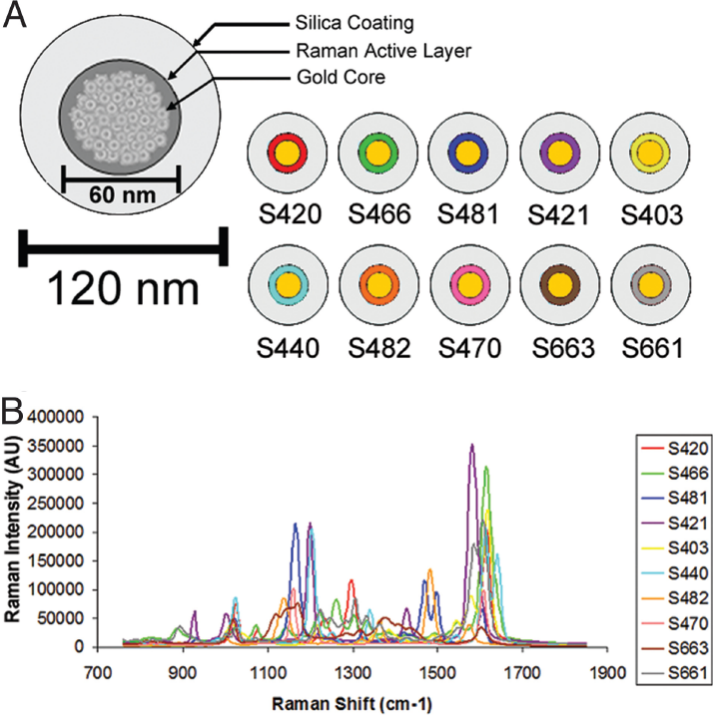
Schematic representation of a SERS Raman nanoparticle and graph depicting unique Raman spectra associated with each of the 10 SERS nanoparticles used for *in vivo* multiplexed imaging. (A) Schematic of a SERS Raman nanoparticle consisting of a 60-nm gold core with a unique Raman active layer adsorbed onto the gold surface and coated with glass totaling 120 nm in diameter. The trade name of each SERS nanoparticle is depicted to the right, where a color has been assigned to the Raman active layer of each SERS nanoparticle. (B) Graph depicting Raman spectra of all 10 SERS nanoparticles; each spectrum has been assigned a color corresponding to its unique Raman active layer as shown in (A). © The National Academy of Sciences.

One way to increase the Raman signal is to use small (10–100 nm size) gold metal spheres or rods. Termed surface-enhanced Raman scattering (SERS) [10], the combination of geometry and materials gives rise to a massive enhancement of the electromagnetic field which can be matched to the laser wavelength. This amplifies the incident laser power like a radio antenna, and also acts like a transmitter to enhance the Raman-shifted signal. Enhancements as high as 10^14^ have been recorded [10] from dye molecules on silver particles, but enhancements of several orders of magnitude is typical. These gold (or silver) particles can be ‘bare’ to enhance the Raman signal of a molecule within a few nanometers of the surface of the particle. However, uncoated colloids in solution would stick together, so they are normally coated with a surfactant which also contributes to the enhanced Raman signal. Alternatively, the surfactant-coated gold particle can be encapsulated in a polymer whose thickness is of tens of nanometers, so any surrounding molecules would not have their Raman signal enhanced by its proximity. Such particles are shown in Figure 5 [11], which all have different molecules as surfactants, hence different Raman spectra.

A similar enhancement effect can be produced at the apex of a sharp gold-coated atomic force microscope tip [12,13]. This tip-enhanced Raman scattering (TERS) microscopy has a resolution of around 10 nm, and imaging on this scale has been performed on carbon nanotubes [14] which are very strong Raman scatterers. Biological molecules have far lower Raman scattering, so several seconds per spectrum is still required. Heating of the gold tip by absorption, limits the usable illumination power to <50 μW because of boiling of a water film around the tip apex [15]. A similar effect can be observed with SERS particles [16–19]—we have observed melting of particles with below 1 mW laser power in a diffraction-limited focal spot.

Another way to achieve an enhancement is with resonance Raman—choosing the excitation wavelength to match an absorption maximum. An example of this is the cytochrome c excited with 532 nm in Figure 4. Most resonance Raman occurs in the near ultra violet (∼200–300 nm) but this is especially damaging to live cells.

In the last decade, Coherent anti-Stokes Raman scattering (CARS) microscopy has been applied to biological samples [20–23]. Instead of using high-frequency electromagnetic radiation (*i.e.*, visible light) to excite a low-frequency molecular vibration, two different laser frequencies are used. The difference between these laser frequencies is matched to the vibrational frequency of the molecule of interest. This resonant excitation produces around 5 orders of magnitude more signal than normal Raman scattering, but is limited to the excitation of only one vibrational frequency rather than a full spectrum. Detailed, high quality images can be acquired in a matter of seconds [23–25]. The CARS signal is proportional to the square of the concentration, so two variants of CARS have been developed to probe low concentrations of molecules: heterodyne CARS [26] and Stimulated Raman loss (SRL) microscopy [27]. Another variant, multiplex CARS, has been developed in order to produce a full spectrum in the millisecond range [28–31]. In multiplex CARS, one of the laser frequencies is replaced by a wide range of wavelengths. CARS has the benefit of speed, whereas Raman has the benefit of full spectral acquisition. Both techniques can be implemented on the same system [32]. A direct comparison between Coherent Stokes Raman Scattering (CSRS, a technique closely related to CARS) and Raman is presented by Cui *et al.* [33].

## 2. *In Vitro* Spectroscopy and Microscopy

It would be impossible to include every published article concerning Raman spectroscopy *in vitro* in this review article. Some examples of results from cells measured ‘*in vitro*’ have already been presented in Figures 2–4, but we will tend to direct the reader to other reviews which concern themselves with different application areas.

Microbial cells are discussed in depth by Harz *et al.* [34], with Raman spectroscopy capable of single cell analysis. Rapid detection of microbial cells (bacteria and yeast) is essential to prevent infection, and Raman spectroscopy provides a near-instant characterization compared to alternative biochemical tests. Raman spectroscopy is able to characterize cell species and strain, as well as live/dead state. Ultraviolet Raman (with excitation in the region of 250 nm) resonantly excites proteins, provides significantly improved signal levels, and can be applied to microbes with far less photodamage than when applied to mammalian cells. A study of ultraviolet resonance Raman spectra applied to bacteria was performed by Nelson *et al.* [35]

The ability of Raman to measure a live/dead state (*i.e.*, cell viability) is applicable to mammalian (eukaryotic) cells. Published work in this area is reviewed by Notingher [36]. Significant differences to spectra relate to changes in the amount of DNA measured, as well as lipids and proteins. This opens up a new method for real time monitoring of toxic agents such as the presented proven examples of Ricin and mustard gas, as well as the effects of chemotherapeutic agents (drugs).

Disease recognition by both Raman and infrared spectroscopies, is reviewed by Krafft *et al.* [37]. Disease causes biochemical changes to cells and tissues, which is measured by these vibrational spectroscopies. The range of diagnosed diseases which is discussed, is extensive—tumors of epithelial tissue, brain tumors, prion diseases, bone diseases, atherosclerosis, kidney stones and gallstones, skin tumors, diabetes and osteoarthritis.

Cancer is second only to heart disease, in terms of cause of death in the developed world. Its early diagnosis is key to reducing the risk of death, and monitoring the response of cancer cells to potential chemotherapeutic agents is crucial to speeding up the selection of new drug candidates. Keller *et al.* [38] have reviewed Raman spectroscopy for cancer diagnosis. The technique is suited to detecting small biomolecular changes that are associated with cancer: an increased nucleus-to-cytoplasm ratio, disordered chromatin, higher metabolic activity, and changes in lipid and protein levels.

Stem cells differentiate into a wide range of cell types, and are likely have a profound effect on medical advances in the 21st century. The greatest challenges [39,40] in regenerative medicine are to ensure high purity of isolated stem cells, and to control the differentiation of stem cells. Both of these issues are addressed by applying non-invasive real-time Raman characterization to stem cells and their derivatives. This topic, along with infra-red spectroscopy, is reviewed by Downes *et al.* [41] and Chan *et al.* [42]. The process of stem cell differentiation is at present monitored by immunocytochemistry [43,44]. However, this process is time consuming as well as requiring the discovery of biomarkers or labels for all cell types.

Denser, more crystalline materials produce a higher Raman signal than molecules in aqueous solution. The application of Raman and infrared spectroscopy to mineralized tissues—*i.e.*, bones and teeth—is reviewed by Carden [45]. Spectroscopy is used to study the mineralization process, to define the changes in chemical structure accompanying bone diseases, and to characterize interactions between tissues and prosthetic implants.

*In vitro* Raman spectroscopy is typically applied to tissue sections, or to live adherent cells on substrates. Floating cells in solution normally move too rarely and too quickly through the focal spot to be characterized with Raman spectroscopy. To trap cells in solution, optical tweezers are used [46]. Tens of milliWatts of power are used to create a strong optical trap based on refraction of light through the transparent cell, and the conservation of momentum. Two separate approaches are used for Raman spectroscopy in these optical traps. The first uses the same beam to trap the cell as for the Raman excitation laser [47], typically at a wavelength of 785 nm. An alternative method is to use separate beams for trapping (typically 1,064 nm) and Raman spectroscopy (typically 785 nm) [48].

Raman spectroscopy publications have been widespread, but Raman imaging is far less common due to the extremely long acquisition time for pixel-by-pixel acquisition. However, very recently, slit scanning Raman imaging has improved the acquisition time greatly, to a matter of minutes. This means that live adherent cells—which move slowly—can be imaged, such as the HeLa cells in Figure 4 [9].

Another way to improve the speed of imaging is to use CARS microscopy, which acquires images in seconds rather than minutes. Its drawback compared to Raman is that it only tuned to one spectral peak rather than acquiring the full spectrum, but it can still be used to acquire images of lipids using the C-H_2_ stretch [21,25], mitochondria using the C-H stretch [21], alpha-helix proteins [49], beta-sheet proteins [9,50], DNA using the phosphate vibration [51], deuterated molecules [52], and collagen and elastin [53].

**Figure 6.**
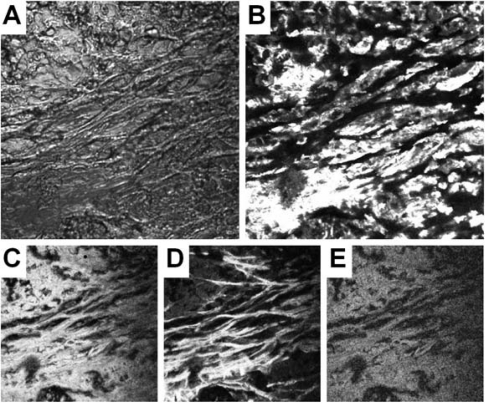
Complementary epi-detected confocal images from the same 212 μm square region of a 20 μm thick sample of cancerous breast tissue. (a) Differential interference contrast (DIC), (b) CARS tuned to 1,662 cm^−1^, (c) two-photon fluorescence (2PEF), (d) second harmonic generation (SHG), (e) sum frequency generation (SFG). Images (b) and (e) were acquired simultaneously, with 11 mW of 1,064.4 nm and 16 mW of 904.4 nm (measured at the sample), all other images were acquired sequentially with illumination only at 904.4 nm. All pixel dwell times were 61 μs (except ‘a’: 1.7 μs), and all images were 512 × 512 pixels. The scan unit dichroic mirror was at 870 nm, the dichroic mirror in the filter block (Photomultiplier detector unit) was at 670 nm. Further short pass filters were applied before ‘b’ (800 nm) and ‘c’, ‘d’, ‘e’ (660 nm). Band pass filters were at 800 nm (width 30 nm) in ‘b’, 535 nm (width 30 nm) in ‘c’, 460 nm (width 50 nm) in ‘d’, 480 nm (width 30 nm) in ‘e’. © John Wiley & Sons, Ltd.

Complementary label-free multiphoton microscopy techniques exist—second harmonic generation (SHG) which highlights non-centrosymmetric ordered structures especially collagen, and sum frequency generation (SFG) which is spectroscopic and sensitive to surfaces and interfaces (where inversion symmetry is broken). In addition, two-photon excitation fluorescence (TPEF) can be acquired, to monitor fluorescent probes and intrinsic autofluorescence of cells and tissue with reduced photodamage and photobleaching. All these properties can usually be imaged with a CARS microscope, as all these multiphoton techniques rely on femtosecond or picosecond laser pulses. Such multimodal systems [23,53–56] are particularly suitable for characterization of tissue. An example of a set of multiphoton images of cancerous breast tissue is presented in Figure 6 [23].

All of the *in vitro* techniques can be performed on cells in their native culture media. In order to preserve signal levels, a high numerical aperture lens should be used—so thin glass-bottom dishes are preferred for CARS imaging. For Raman, thin quartz cover slips are available—these can be used as the bottom part of a dish in a cover slip kit. Many cell types grow well on plastic, but do not adhere well to glass or quartz. So it is common to coat substrates with collagen or gelatin, or for stem cells, Matrigel™ (BD Biosciences) or CellSTART™ (Invitrogen).

## 3. *In Vivo* Spectroscopy

**Figure 7.**
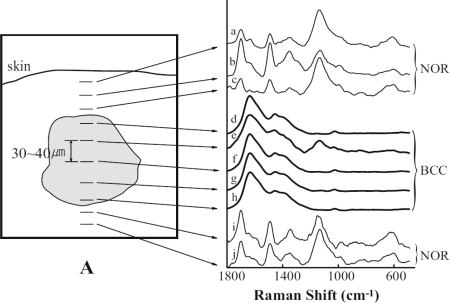
Confocal Raman profiles of skin tissue with an interval of 30–40 μm. © Springer-Verlag.

The penetration depth for visible light through tissue is limited by both absorption (which decreases with wavelength) and scattering (which increases with wavelength), to a few millimeters. Hence the use of Raman spectroscopy and CARS microscopy for non-invasive characterization is limited to the skin and dermal layers. A Raman spectroscopy system for dermal characterization is described by Caspers *et al.* [57]. Skin cancers such as melanoma and basal cell carcinoma can be diagnosed simply, as is shown in Figure 7.

CARS microscopy has also been applied to skin layers [25], and Stimulated Raman loss (SRL) microscopy has acquired images tuned to the vibrational frequency of dimethyl sulfoxide (DMSO) which is a vehicle for transporting drug molecules through the skin [27].

Scattering by tissue causes the focal spot to broaden as a function of depth below the skin. As CARS microscopy requires three photons to be absorbed, and the CARS signal depends inversely on the third power of the spot size, its penetration depth is reduced to the sub-millimeter scale. However, adaptive optics [58] has increased this penetration depth by shaping the excitation beams to compensate for both the optical system of the microscope and aberrations caused by refractive index changes in the tissue.

**Figure 8.**
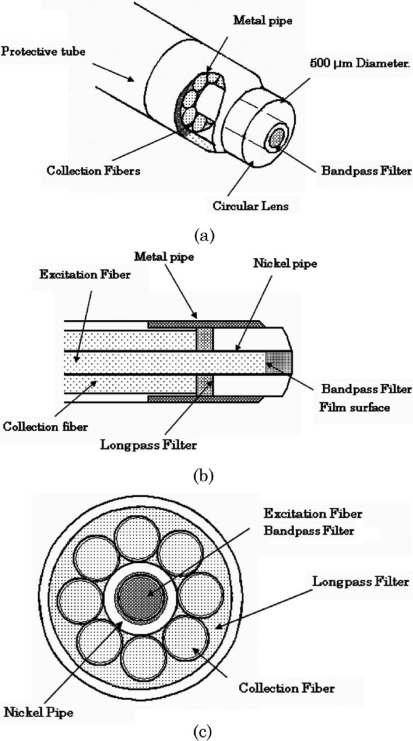
(a) Structure of distal end of a micro Raman probe (MRP). (b) Longitudinal cross section of distal end of a MRP. (c) Transverse cross section at fiber-filter interface. © 2009 Optical Society of America.

Raman spectroscopy can also be used invasively *in vivo* by combining with endoscopy. Using a single fiber to transmit the laser and collect Raman-shifted light, is not effective because Raman transitions from the glass of the optical fiber dominate over any signal acquired from tissue. One way of circumventing this problem is to use separate fibers for excitation and collection. A notch or short pass filter must also be placed after the excitation fiber to remove all Raman-shifted light generated in the excitation fiber, to leave pure laser light. In addition, a long pass filter must be placed in front of the collection fiber, to remove all the laser light but retain the Raman signal of interest from the tissue. The ideal fiber system, drawn in Figure 8, consists of a single excitation fiber surrounded by six collection fibers [59]. This allows for a high collection efficiency while maintaining a small diameter required for keyhole surgery. Such small fiber bundles have been used in research to characterize artery walls [60,61]. However, commercially available probes are far larger—typically 10–12.7 mm in diameter, so can only be used during open surgery or possibly for oral or rectal examination.

Raman spectroscopy has also been applied to ophthalmology, and a comprehensive review by Erckens *et al.* [62] charts its progress to date. A more recent study [63] uses Raman spectroscopy to measure ocular aging.

**Figure 9.**
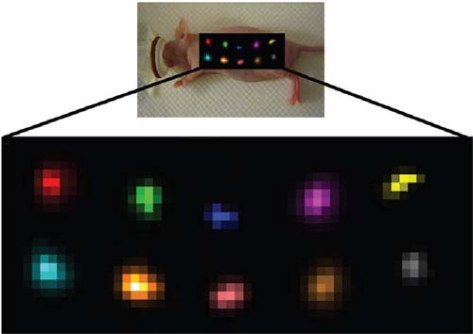
Evaluation of multiplexing 10 different SERS nanoparticles *in vivo*. Raman map of 10 different SERS particles injected subcutaneously. in a nude mouse. Arbitrary colors have been assigned to each unique SERS nanoparticle batch injected. Panels below depict separate channels associated with each of the injected SERS nanoparticles. Grayscale bar to the right depicts the Raman intensity, where white represents the maximum intensity and black represents no intensity. The postprocessing software was able to successfully separate all 10 SERS nanoparticles into their respective channels with minimal crosstalk. © The National Academy of Sciences.

**Figure 10.**
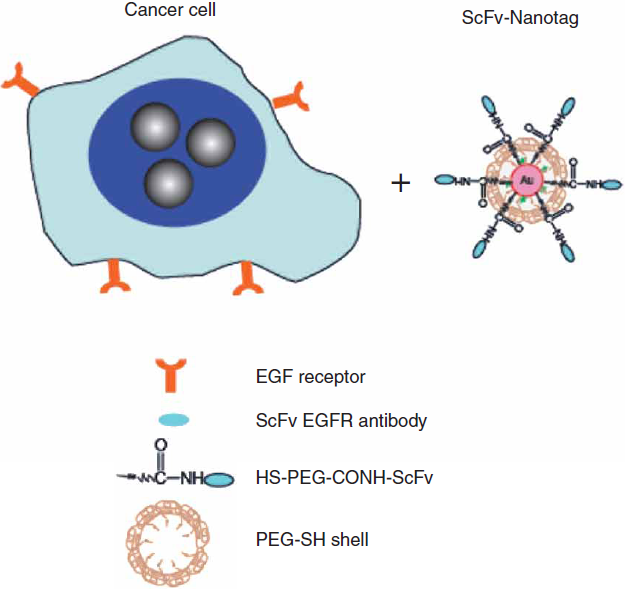
Cancer cell targeting and spectroscopic detection by using antibody-conjugated SERS nanoparticles. Preparation of targeted SERS nanoparticles by using a mixture of SH-PEG (thinly—polyethylene glycol) and a hetero-functional PEG (SH-PEG-COOH). Covalent conjugation of an EGFR-antibody (epidermal growth factor receptor) fragment occurs at the exposed terminal of the hetero-functional PEG. © 2010 Nature Publishing Group.

**Figure 11.**
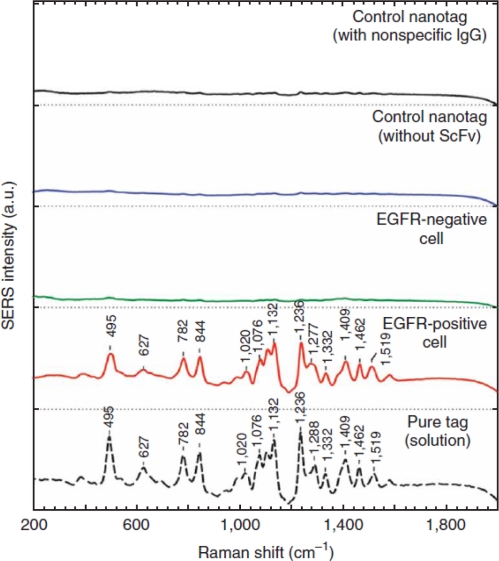
SERS spectra obtained from EGFR-positive cancer cells (Tu686) and from EGFR-negative cancer cells (human non-small cell lung carcinoma NCI-H520), together with control data and the standard tag spectrum. All spectra were taken in cell suspension with 785-nm laser excitation and were corrected by subtracting the spectra of nametag-stained cells by the spectra of unprocessed cells. The Raman reporter molecule is diethylthiatricarbocyanine (DTTC), and its distinct spectral signatures are indicated by wavenumbers (cm^−1^). © 2010 Nature Publishing Group.

Surface enhanced Raman spectroscopy (SERS) has been applied to polymer-coated gold particles containing many different molecular markers (shown in Figure 5). These SERS particles have been injected into animals, then in Figure 9 a spectrum was acquired at a number of imaging pixels to reveal their location. Due to the enhancement of the gold particle, concentrations as low as 100 pM were observed. These particles can then be conjugated with antibodies to diseased cells [64]. In Figure 10, particles were coated with antibodies to a particular type of lung cancer, and *in vitro* experiments in Figure 11 revealed that the particles adhered to these cells but not the control sample [64]. This means that these particles could compete with existing techniques for cancer diagnosis—positron emission tomography (PET) imaging using a radioactive isotope of fluorodeoxyglucose (18F-FDG) which accumulates in tumors, and magnetic resonance imaging (MRI) which uses gadolinium and magnetic particle labeling. These Raman labels will only be visible within millimeters of the skin (or in open surgery, ophthalmology, or with endoscopy) so have limited use in medical diagnosis of diseases. They do, however, have great potential in understanding cancer and drug trials by using small animals. Raman labels have superior characteristics to fluorescent labels, which are prone to photobleaching and may be drowned out by autofluorescence of tissue. But the major advantage is the ability to multiplex a large number of these tags, unlike the limit of 3 or 4 which can be used together in fluorescence imaging.

## 4. Statistical Analysis

A raw, unprocessed Raman spectrum is shown in Figure 2. As well as the sharp spectral peaks, spectra typically contain some autofluorescence from cells or tissue, which is a very broad peak as wide as the whole Raman spectrum. This can be removed by subtracting a polynomial, in a process known as baseline subtraction, to ‘flatten’ spectra into those such as in Figures 3, 5, and 11.

In order to extract all information from the Raman spectrum or Raman image, some form of statistical analysis is required. The most simple change to a spectrum between two cell types, e.g., a diseased/healthy state, or viability (live/dead), would be a change in the intensity of just one spectral peak. However, this is rarely observed—typically, the intensity of several peaks will increase, while several others will decrease.

A supervised procedure for determining a cell type is to first acquire a spectrum from each of a number of cells or tissue of one known type (e.g., cancerous), and a spectrum from a number of cells or tissue of a different known type (e.g., healthy). The cell type can either be known intrinsically, or be measured later with a biochemical assay. These measurements form a training set. Then an unknown cell is put into the spectrometer in order to determine its cell type, by its similarity to one or other sets of spectra from the two (or more) known cell types. A series of spectra from unknown cells represents a test set, which can be used in determining the accuracy of the prediction.

An alternative method is an unsupervised procedure, which characterizes a group of cells of unknown cell type. All these spectra can be plotted as individual data points on a graph with co-ordinates which attempt to highlight the greatest differences in the spectra of individual cells or tissue samples. The user can then ascribe how separated are the groups of spectra, but it cannot be judged which group is which (e.g., healthy or cancerous).

**Figure 12.**
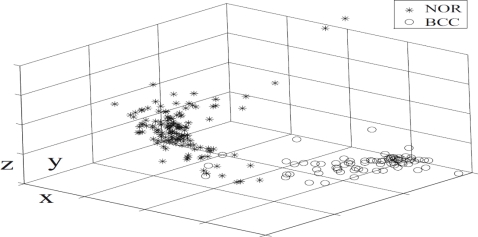
Distribution of the first three components of PCA transformed spectra (of biopsy samples from 10 patients) © Springer-Verlag.

The most common method of spectral analysis is principal component analysis (PCA) [65]. This transforms the set of spectra to a new coordinate system. Each acquired spectrum is transformed into a set of basis spectra (or, co-ordinates), so each acquired spectrum can be reconstructed from a mixture of proportions of this basis set of spectra. The first spectrum of this basis set, called the first principal component, gives the greatest variance (*i.e.*, difference) between all the acquired spectra. The proportion of this first principal component spectrum present in each of the acquired spectra, can be plotted on one axis of a graph. The second principal component contains the second greatest variance between all acquired spectra, and the proportion of this spectrum present in each of the acquired spectra is plotted on the second axis of the graph. Such a graph of these first two or three principal components—such as the one in Figure 12 [57], with spectra acquired in the same way as Figure 7—is usually enough to sort out all acquired spectra (each represented by a data point on the graph) into separate regions, though there is some overlap between sets of spectra.

Cluster analysis [66] is an unsupervised technique applied to a set of Raman spectra which have already been treated with PCA. This set of spectra can either be from a series of cells or tissue samples, or be from an array of pixels in a Raman image. K-means cluster analysis [67,68] sorts all the data points (*i.e.*, spectra) into a predetermined number, k, of groups. Initially, random points are chosen on the PCA graph as centroids of the groups, then each data point is assigned to the nearest of these centroids. When all points have been assigned into groups, new centroids are defined as the average of all spectra in the groups. This process is repeated until all points remain in the same groups. An example of a Raman image classified into different regions using k-means clustering, is shown in Figure 3.

Hierarchical cluster analysis (HCA) [69] is a more rigorous cluster analysis. The distances between every pair of points within the total number of n points, is calculated and into an (n × n) matrix. The two points which are closest together are found, and assigned as the first cluster. This one cluster and the remaining points are treated as n–1 objects, and closest two objects are assigned as the second cluster. This process is repeated n–1 times until all points are grouped into one single cluster of points. The outcome is a tree-like diagram, where data points can be grouped into any required number of groups.

## 5. Conclusions

Raman spectroscopy is an extremely powerful technique for characterizing biological systems. Simple spectroscopy can be used to diagnose disease in cells and tissue even without understanding the mechanism or biochemistry involved, and it helps the understanding of basic biology when we can interpret the changes in spectra between samples. Raman imaging had until recently been restricted to fixed cells and tissue, but two improvements—slit scanning Raman microscopy, and CARS microscopy, have speeded up image acquisition to minutes and seconds respectively. This means that live cells can be imaged, thereby enhancing the biology which can be studied.

*In vivo* Raman spectroscopy for medical diagnosis is either restricted to ophthalmology, or to a relatively thin layer of skin, or during open surgery, or in conjunction with miniaturized endoscopic probes can be used with minimally invasive surgery. SERS particles act as Raman markers, which have great potential in diagnosis of diseases, but are limited to small animal research studies, or in conjunction with endoscopy.
